# Opioid withdrawal syndrome‐associated cognitive impairment: A narrative review

**DOI:** 10.1111/ajad.70201

**Published:** 2026-09-17

**Authors:** Faizan Khan, Nazli Tunali, R. Andrew Chambers

**Affiliations:** ^1^ Addiction Psychiatry Program, Department of Psychiatry Indiana University School of Medicine Indianapolis Indiana USA

## Abstract

**Background and Objectives:**

Opioid withdrawal syndrome (OWS) comprises a transient constellation of subjective symptoms and objective signs expressed by brain and other organs. Although cognitive impairment is well documented in opioid use and addiction, little is known about cognitive impairment that may specifically accompany OWS. This paper explores accumulating literature describing Opioid Withdrawal Syndrome‐associated Cognitive Impairment (OWSCI) which may inform clinical care and therapeutics research.

**Methods:**

A search of studies published before August 2026 was conducted via PubMed and Google Scholar (309 and 626 papers screened) via crossing “cognition” with “opioid withdrawal,” “early opioid abstinence,” “opioid use,” or “opioid addiction.” Bibliography searches were also conducted of core target papers defined as human adult studies measuring cognition during OWS from 1 to 15 days after last opioid use.

**Results:**

Only seven studies were identified meeting core criteria. These varied considerably by design, patient samples, comorbidities, timing and methods of cognitive testing, and OWS treatments. The evidence thus far suggests OWSCI is a measurable transient feature of OWS that overlays, and is modulated by, a wide range of clinical variables including opioid history, medication treatments, other addictions, and neuropsychiatric comorbidities.

**Discussion and Conclusions:**

Future studies on OWSCI entailing more rigorous experimental controls and standardizations, while accounting for diverse comorbidities and OWS treatments, are needed to more fully characterize OWSCI.

**Scientific Significance:**

Growing data on OWSCI are deepening our understanding of neurobiological and clinical dimensions of OWS, potentially providing clues for improving treatments for patients transitioning through OWS into opioid‐free recoveries.

## Abstract 2

Answer questions and earn CME credit

## INTRODUCTION

Opioid addiction and mortality are major public health concerns especially among patients with mental illness.^1^ Opioid use disorders (OUDs) are a spectrum of treatable conditions spanning intoxication, opioid withdrawal syndrome (OWS), and addiction that involve differential sensory, emotional, motivational, and cognitive symptom components.^2^ A focus on newly characterized neurobiological and clinical aspects of OWS,^3^ not necessarily described in traditional OWS criteria (e.g., in DSM‐5)^4^—such as cognitive symptoms—may provide new insights for neurobiological, clinical, and therapeutics research for OUDs.

Although numerous studies have examined cognitive deficits associated with opioid intoxication or addiction,^5^, ^6^ relatively few studies—and to our knowledge no reviews—have compiled evidence on cognitive impairment that may occur during or specifically resulting from acute OWS (termed here as OWS‐associated Cognitive Impairment [OWSCI]). This review summarizes available evidence pertaining to these questions: How might OWS produce cognitive deficits (i.e., potentially superimposed on cognitive impairments associated with other components of the OUD spectrum) and what is their quality, severity, and timing? Could OWSCI be significant enough to be considered in future DSM criteria for OWS (in addition to the autonomic, somatic, and dysphoric criteria currently listed)^4^? Researching answers to these questions may provide clues about neurocircuit dysfunction occurring in OWS and opioid addiction informing novel treatment development. In everyday clinical circumstances, patients in deep OWS may be simultaneously challenged with various personal, legal, and medical issues or treatment decisions associated with sudden significant declines or cessation of opioid use. This could at least hypothetically happen when missing appointments in opioid maintenance treatment, being suddenly hospitalized (e.g., due to injuries, medical illness, or overdose), experiencing transitions in opioid addiction care, or incarceration. These contexts require cognitive capacities of patients, and awareness of clinicians about cognitive compromise during OWS. In terms of treatment and therapeutics research, it is possible that OWSCI could impact patients' decision‐making to stay in versus abort a detoxification protocol or inpatient stay. Also, it may be important to understand if medications that treat OWS could exacerbate or reduce OWSCI, and how such effects could enhance OWS treatment. Thus, an appraisal of existing literature characterizing OWSCI may inform and motivate future studies on OWSCI, providing clinical and neuroscientific implications that could improve OWS care.

## METHOD OF LITERATURE REVIEW

Our search for evidence in the core topic domain sought primary research articles published prior to July 31, 2026, appearing in PubMed and Google Scholar that test cognition in patients undergoing OWS. Our primary search crossed the key terms “opioid withdrawal” and “early opioid abstinence” with “cognition.” We also crossed “opioid use” and “opioid addiction” with “cognition” as secondary searches in PubMed and Google Scholar, to estimate the sizes of the literature studying cognition in these other contexts in comparison to the volume of returns on cognition in OWS (and to secondarily check for possible target OWSCI reports or reviews there). The numbers of publications/reports returned in these searches are shown in Table 1. Our PubMed searches based only on these crossed terms (with no filters) returned sets that were orders of magnitude smaller (309 publications) than in Google Scholar without filtering (~30,000, not shown in Table 1). So, for the PubMed search, we reviewed all 309 abstracts manually, seeking (a) primary research papers in (b) English language, focused on (c) human and (d) adult subjects with (e) methods clearly stating they measured cognition at least once in patients from 1 to 15 days after last use of any and all opioids (including maintenance medications [e.g., methadone/buprenorphine (BUP)]). In contrast, for the Google Scholar search, we built specific limits into the search, using terms that would substantially exclude review articles (‐review), animal modeling (‐animal), and neonatal studies (‐neonatal), returning the publication numbers shown in Table 1 under Google Scholar. Then for this set, we manually screened all 626 abstracts returned from crossing “cognition” with “opioid withdrawal” and “early opioid abstinence.” There were no review papers found on OWSCI anywhere. Relevant papers found as being close to, or within the core topic domain (criteria a‐e above) also had their bibliographies searched for additional sources not found in the electronic databases.

**Table 1:** Search method results.

Terms crossed in search	PubMed	Google Scholar ‐review, ‐neonatal, ‐animal
*“opioid withdrawal” × “cognition”*	25*	624*
*“early opioid abstinence” × “cognition”*	10*	2*
*“opioid use” × “cognition”*	248*	4240
*“opioid addiction” × “cognition”*	26*	1090

*Note*: Numbers of abstracts/publications generated by search term crosses per search engine. *Denotes abstracts/publication sets that were manually perused for possible target articles meeting core search criteria focused on cognitive testing in opioid withdrawal syndrome (OWS) ≤ 15 days from last opioid use. PubMed numbers reflect simple search term crosses (with no exclusions built into the automated search); exclusions of the PubMed sets were conducted manually by the authors. Google Scholar numbers reflect results with exclusions already built into the automated search: screen out review articles (‐review); neonatal studies (‐neonatal); and animal models (‐animal).

Our adoption of criterion (e) requiring 1–15 days since last opioid use was implemented based on the following: OWS varies greatly between individuals showing onset, peak intensities, durations, and clinical symptoms that differ based on patient characteristics (e.g., metabolism) and opioid history (e.g., type, dosing, durations, pharmacokinetics, and potency).^2^ Even with this heterogeneity, there is consensus that OWS (after total abstinence) generally begins, peaks, and mostly resolves within 2 weeks of last opioid use.^2^ Moreover, the recommended guidelines for opioid antagonist treatment for opioid addiction (e.g., with naltrexone [NTX]), which can produce significant precipitated withdrawal if the patient is still in substantial natural OWS, is 7 plus days since last opioid use (of any kind).^7^ Also, through our search, we noted that many studies in the cognition‐OUD literature do not attend to OWS, do not clearly indicate if or when patients are still in OWS, or if they are in a state of partial‐minimized withdrawal while still taking opioids, as in a detox‐taper. So, we used the fairly reliable and straightforward criteria of requiring our core papers to state that patients were 1–15 days out from their last opioid use when undergoing at least one session of cognitive testing. According to these criteria, and for the purposes of this review (and interpreting Tables 2 and 3), we somewhat arbitrarily labeled early OWS as 1–2 days from last opioid use; mid‐OWS as 3–7 days since last use, and late‐OWS as 8–15 days since last use. Our search strategy and reporting on the relatively older and yet underdeveloped literature on OWSCI was informed by recently endorsed guidelines for Scoping^8^ and Narrative reviews.^9^ Below, we provide a brief clinical and scientific contextualization of the OWSCI literature, followed by a description, summary, and discussion of the core target papers identified.

**Table 2:** Characteristics of Opioid Withdrawal Syndrome‐associated Cognitive Impairment (OWSCI) studies.

First author/year	Participants/study goal	Design/OWS treatment	Study set characteristics (inclusion/exclusions)	Innovation/drawback	Measures of OWS/timing of cog testing during OWS	Results support Existence of OWSCI?	
Guerra 1987	***n* = 93** (age 18–35; 28 female) Spanish patients with heroin addiction, detoxed. **Measure cognition while in use then in OWS, compare to HCs (*n* = 30)**	**Repeated measures design and cross‐sectional comparison**: Test while in use and then 7–14 days after start of 7–10‐day **detoxification taper with CLD or MTD**	History of heroin addiction with comorbid personality disorders, mood disorders, and other use disorders (sedatives, alcohol)	First study to examine for OWSCI Many poor standardizations	**OWS not measured.** 2 cognitive tests per patient, 1 for HC; Cog testing in **early to late** **OWS** (7–14 days after start of 7–10‐day detox)	Inconclusive. Cognition in OWS better than in prior *uncontrolled* opioid use	
Kanof 1992	***n* = 20** male (age 23–58; 0 female), US veterans with heroin addiction, stabilized on MTD, 24 mg/day. **Measure cognition during acute NLX‐precipitated OWS**	**Repeated measures cross over design:** Following a week of MTD maintenance, test after iv NLX versus PBO delivered 2 days apart. Cognition assessed 10 min post NLX versus PBO. **No OWS treatment**	No substantial daily use of alcohol or non‐opioid drugs; no mental or medical illness	First study to examine OWSCI in precipitated OWS Precipitation brief/shallow	**SOWS, OOWS** Acute precipitated OWS, tested 10 min after NLX/PBO and approx. 24 h since last MTD). 2 cognitive tests per patient (acute **early OWS** vs. during MTD maintenance)	No	
Lyvers 2003	***n* = 39** (age 18–45; 21 female) Australian outpatients, stable on MTD, >25 mg/day. **Measure cognition in early OWS versus just dosed MTD patients**	**Cross‐sectional 2 group comparison**: Test in early OWS (*n* = 18) (24 h since last MTD) versus just dosed (*n* = 21) (90 min post dose). **No OWS treatment**	Consumption of <14 alcoholic drinks per week; no recent use of CNS‐active drugs other than methadone, no prior alcohol or other use disorders; no major medical/psychiatric history	First study to examine OWSCI in methadone maintenance Many exclusions unnaturalistic	**OWS not measured** 1 cognitive test per patient in **early OWS**	YES Cognition in early OWS worse than just Dosed in stable Methadone treatment	
Rapeli 2006	***n* = 15** (age 20–50; 4 female) Finnish patients with unspecified OUD type entering inpatient detox. **Measure cognition in mid‐ to late‐OWS**	**Cross‐sectional 2 group comparison**: Testing performed 5–15 days post last opioid delivery versus HC (*n* = 15). OWS treatment various including BZDs, LFX, no opioids	No “uncontrolled” poly substance use, recent heavy alcohol use, or acute psychiatric illness (9 patients with co‐occurring BZD dependence, 2 with CB dependence)	First study to examine OWSCI in relation to time since last opioid use Confounded by OWS treatments	**ShOWS** 1 cognitive test per patient in **mid‐ to late‐OWS**	YES Cognitive deficits in mid‐late‐OWS compared to HCs, improve in OWS with time since last use	
Saroj 2020	***n* = 90** male (mean age 28 [SD 7]; 0 female) Indian patients with OUD (unspecified) on maintenance treatment or early OWS. **Measure cognition in OUD patients as a function of being on BUP, NTX, or early OWS versus HCs**	**Cross‐sectional 4 group comparison:** Test in BUP (*n* = 20) > 6 months stable; NTX (*n* = 20) > 6 months stable; OWS (1–4 days since last use (*n* = 20), HCs (*n* = 30). **OWS treatment anchored on CLD**	OUD patients in BUP or NTX groups > 30 days since last illicit OP use; OWS patients (mean 2.2 days since last use), no moderate to severe OWS (COWS > 12 excluded). No current other SUDs (other than nicotine); no major mental or medical illness	First study to examine OWSCI in comparison to multiple other treatment status groups Repeated measures with multiple groups not done	**COWS** 1 cognitive test session per patient; testing **in early‐mid‐OWS**		YES Cognition worse overall in OWS group, compared to BUP/NTX patients, HCs (no OUD history) with best performance
Kaur 2023	***n* = 50** male (age 18–55; 0 female) Indian outpatients with OUD (unspecified) **Measure cognition within subjects across stages of OWS**	**Repeated measures:** Test before (BL), at late‐OWS (2 weeks since last use), and after OWS (8 weeks). **OWS treatment anchored on CLD and ZOL as transition to NTX**	OUD patients with no other SUDs (other than nicotine); no major mental or medical illness; no severe OWS (COWS > 25 excluded)	First study to test across multiple OWS time points and replicate cognitive and OWS measures Detox confounds	**COWS** 3 cognitive test sessions per patient; **before, late‐OWS and after OWS**		Yes Cognitive impairment in active uncontrolled use worse but generally resolving increasingly across late‐OWS to after OWS
Grant 2024	***n* = 53** (age 23–46; 6 females), Russian inpatients with HIV using heroin, MTD, or both, on inpatient detox service **Measure cognition, mood, stress, and inflammatory markers in HIV patients undergoing OWS**	**Repeated measures**: Test at early OWS (Day 1 inpatient), mid‐late (Days 7–14), and after OWS (3–5 weeks post last use). **OWS treatment anchored on CLD**	Advanced HIV, no prior or current antiretroviral therapy, currently psychotic, suicidal or homicidal ideation. Other SUD not excluded but patients did not have recent cannabinoid or cocaine use	First study to examine OWSCI with Biomarker in multiple OWS time points Untreated HIV sample may be ungeneralizable	**COWS, SOWS** 3 cognitive test sessions per patient; **early, late, and after OWS**		YES Cognitive impairment in early OWS improves to after OWS; with some non‐significant trends for mid‐OWS cognition worse than early OWS. Cognitive impairment associates with HPA activation

Abbreviations: BL, baseline; BUP, buprenorphine; BZD, benzoid/benzodizepine; CB, cannabinoid; CLD, clonidine; CNS, central nervous system; COWS, Clinical Opioid Withdrawal Scale; HCs, healthy controls; HIV, human immunodeficiency virus; LFX, lofexidine; MTD, methadone; NLX, naloxone; NTX, naltrexone; OOWS, Objective Opioid Withdrawal Scale; OP, opioid; OUD, opioid use disorder; OWS, Opioid withdrawal syndrome; PBO, placebo; ShOWS, Short Opioid Withdrawal Scale; SOWS, Subjective Opioid Withdrawal Scale; SUD, substance use disorder; ZOL, zolpidem.

**Table 3:** Cognitive testing and findings across Opioid Withdrawal Syndrome‐associated Cognitive Impairment (OWSCI) studies.

First author/year	Reported neurocognitive Cognitive test name	Reported neurocognitive Functional correlates per test	Key findings indicating OWSCI
Guerra 1987	**Toulouse‐Pieron (TP)** **PMA‐F** **MAI‐Digit Span** **Memory of words** **Evocation of events**	Attention* Verbal fluency* Immediate recall* Short‐term memory* Long‐term memory*	Cognition improved (no relative deficit, during OWS; stage not defined) compared to prior active uncontrolled heroin use
Kanof 1992	Stroop Color & Word Test Digit Span (Wechsler)	Visual perception/naming Attention, immediate recall	Cognition not changed in mild early precipitated OWS
Lyvers 2003	Wisconsin Card Sort	**Executive functioning,** **Cognitive flexibility,** Response inhibition*	**Cognition impaired** in early OWS, compared to MTD maintained
Rapeli 2006	Digit Span (Wechsler) **PASAT** Delayed Logical Memory Rey Auditory Verbal Learning Test Benton Visual Retention Test Modified Stroop Task **Ruff Figural Fluency Test** **Culture Fair Intelligence Test**	Verbal working memory Complex Working Memory** Verbal memory Verbal memory Verbal memory Executive function Executive function* Fluid Intelligence**	**Cognition selectively impaired** in OWS versus HC in mid‐late‐OWS; **performance positively correlates with longer duration from last opioid use
Saroj 2020	**TMT** **DVT** COWA **Verbal N‐back** **Visual N‐back** Wisconsin Card Sort RAVLT	Mental speed* Sustained attention* Phonemic fluency Verbal working memory* Vis‐spat working memory* Set shifting, working memory, Executive functioning **Verbal learning and memory**	*Cognition variously/selectively impaired in OWS group compared to one or more comparison groups (BUP vs. NTX maintenance vs. HC)
Kaur 2023	**Wisconsin Card Sort** **Digit Span** **Recognition test** **Verbal retention**	**Executive functioning,** **Shifting strategies** **Abstract reasoning** Response inhibition* Attention, concentration* Nonverbal memory* Verbal memory*	*Cognition improved during OWS compared to active use, but **more impaired in OWS** compared to after OWS resolved
Grant 2024	Digit Symbol (Wechsler) WAIS‐III Color Trails Test **PSAT‐50** Hopkins Verbal Learning Test Brief Visuospatial Memory Test Color Trials Test **Category and Action Fluency** **Grooved Pegboard Test**	Attention, processing speed* Verbal memory Visual memory Executive functioning Verbal fluency* Motor speed coordination*	*Cognition relatively impaired in early OWS versus post OWS; some trends for worsening memory from early to mid‐OWS but not significant

*Note*: Cognitive tests shown with (*) **and in bold** were associated with statistically significant findings pertaining to OWSCI, albeit involving quite different comparisons across studies. The names of cognitive tests and forms of neurocognitive functions that these tests were described as assessing are reported here as originally stated by those authors. Readers should view the original papers for relevant citations, specific findings, and their interpretations.

Abbreviations: BUP, buprenorphine; HC, healthy control; MTD, methadone; NTX, naloxone; OWS, Opioid withdrawal syndrome.

## CONTEXTUALIZATION AND COMPLEXITY OF COGNITIVE RESEARCH IN THE OUD SPECTRUM

Cognitive research in substance use disorders (SUDs) has generally been interested in the effects of acute substance intoxication, or the cumulative‐chronic effects of drug use (i.e., in addiction) to produce cognitive impairment.^10^ Conversely, cognitive research on the withdrawal phase of some SUDs, particularly opioids, has received much less systematic study (see Table 1) even though opioid withdrawal, like intoxication, can produce significant psychiatric and somatic signs and symptoms. Substance withdrawal is the second phase of a dynamic neurobiological process that for each drug type resembles the inverse of what intoxication produces.^11^ As with the pharmacological actions and rapid neuroadaptive events that occur when psychoactive drug levels are rising or maintained during intoxication, the brain is also functioning in a physiologically unbalanced state during withdrawal when drug levels are plummeting or gone.^12^, ^13^ In withdrawal, the initial neuroadaptive changes instigated during acute intoxication, especially those produced by chronic drug use, are now unopposed by the drug, keeping the brain off balance, albeit in a somewhat opposite direction from intoxication.^2^, ^13^ This lack of normative neurophysiological balance, and not merely the direction of it in intoxication versus withdrawal, may be key to understanding why and how cognitive deficits could occur in withdrawal as well as intoxication, as has been documented across several addictive drug classes. Thus, cognitive impairments do occur during withdrawal from several types of drugs, regardless of their stimulant versus depressant‐like profiles, including nicotine,^14^ cocaine,^15^ and alcohol.^16^ Still, this literature is pervaded by uncertainty about the extent to which long‐term cognitive impairments associated with prior addiction(s) persist into, or are exacerbated by withdrawal syndromes.^17^, ^18^

In consideration of these and other variables that may contribute to OWSCI, it is important to briefly summarize current knowledge about impairments in different phases of OUD, as they may overlap or compound OWSCI. A full accounting of evidence for cognitive abnormalities across the OUD spectrum is beyond the scope of this review. However, acute opioid intoxication is well known to produce cognitive impairment in association with the central nervous system (CNS) depressant and sensory effects of acute mu‐opioid receptor agonism.^6^ Opioid addiction is also well associated with cognitive deficits, independent of acute intoxication^19^, ^20^, ^21^ as OUD patients also suffer with high frequencies of a wide range of comorbid conditions also known to produce cognitive problems.^22^ Psychiatric disorders associated with OUD including PTSD, psychotic, mood, and ADHD disorders are all associated with cognitive impairments^1^, ^23^ as are commonly comorbid addictions, involving alcohol, benzodiazepines, and marijuana.^10^, ^24^ Further, a range of neuropsychiatric conditions occurring at high prevalences in OUD including Traumatic Brain Injury (from accidents, assaults, overdose‐related hypoxic brain injury, and neural‐thromboembolic events with endocarditis), and HIV can underpin cognitive dysfunction in OUD patients.^25^ So, research examining OWSCI should be expected to be complicated by many possible causes and forms of cognitive impairment associated with having OUD.

Repeated measures of cognition in the same patients as they progress through OWS would be key for teasing apart different forms and causes of OWSCI, providing greater interpretative value than cross‐sectional studies. But even repeated measures studies can be confounded by co‐occurring dynamics, particularly involving intoxication or withdrawal states of drugs other than opioids. Patients attempting an outpatient “detox” often substitute with other drugs while experiencing OWS,^26^ whereas inpatients may experience withdrawal and related cognitive impairment not just from opioids, but other drugs they regularly use, but cannot access while admitted.^27^ Both outpatient or inpatient populations experiencing OWS may also be treated with medications bearing their own cognitive impairing effects, including benzoids (i.e., benzodiazepines, atypical‐benzodiazepines, and barbiturates), gabapentin, and antihistamines. Awareness of these complexities is valuable in assessing the methodological strengths and interpretation of studies attempting to measure OWSCI.

## BASIC NEUROSCIENCE OF OWSCI

Animal modeling allows a degree of experimental control that is elusive in human research. In rodent models, relatively unconfounded OWSCI has been described. For instance, mice show acute cognitive impairments at the initiation of opioid exposure (e.g., with acute intoxication) that resolves with chronic opioid delivery and tolerance.^28^ Then, when the animals undergo precipitated OWS after chronic exposure, they show new cognitive impairment during withdrawal that is more pronounced if the animals have prior histories of repeated bouts of precipitated OWS.^28^ Triggering interest in the mechanisms of this effect, Tramullas et al. examined the role of molecular pathways governing neuronal plasticity and neuronal cell death, that, downstream from the mu‐opioid receptor, are variously pushed and pulled by cycles of intoxication and withdrawal.^28^ These investigators hypothesize a kindling‐like effect of neural injury could happen in patients deeply affected by opioid addiction as is suspected in alcohol addiction patients who experience severe, repeated cycles of intoxication and withdrawal.^29^

Still, multiple other neurobiological mechanisms could underpin OWSCI. OWS is a brain and body stress test that invokes the HPA axis and corticotropin releasing hormones, secondarily impacting inflammatory and immune response systems,^12^, ^30^, ^31^ affect,^2^ and cognition.^32^ Hence, many OWS symptoms mimic those occurring with infections like influenza or COVID. OWS has also long been understood as representing a state of sympathomimetic overactivity, corresponding to agitation, anxiety, insomnia, elevations in heart rate, blood pressure, respirations, and pupillary dilation—all consistent with the fight or flight response.^2^, ^12^ Hence alpha‐2 agonists clonidine and lofexidine, both anchors of modern OWS treatment, block parts of the CNS‐fight or flight reaction, which during OWS, is mediated by elevated norepinephrine release by the locus coeruleus.^33^ Still, more broadly in the brain, OWS also entails a state of overactivity of excitatory glutamate neurotransmission.^34^, ^35^ This excitatory overdrive, along with HPA axis reactivity, sympathomimetic overflow, and changes in endogenous cannabinoid and orexin‐mediated regulation of neurovegetative functions,^31^, ^35^ may all contribute to impaired cognition during OWS, similar to what happens in an acute traumatic experience.^36^

## CORE EVIDENCE FOR OWSCI

Using the search strategy above, our applied target criteria yielded an OWSCI literature of seven papers with publication dates spanning four decades. We describe these below in chronological order, which highlights their evolving methodological sophistication. Key summary data for these studies appear in Table 2 (study characteristics) and 3 (cognitive measures and findings).

### The initial foray: Guerra et al. (1987; Spain)

While cognitive studies in OUD patients first emerged in the mid‐1970s to early 1980s,^5^ Guerra et al. were the first to examine cognition in OWS in 1987.^37^ They used a repeated measures design where subjects were tested concurrent with heroin use, then again after initiating detox, somewhere between early and late‐OWS. This study also provided a cross‐sectional comparison with healthy controls. This study examined cognition during OWS as a control for assessing cognition during active use as a strategy for distinguishing the cognitive effects of ongoing opioid use apart from opioid addiction. In essence, the study was not necessarily hypothesizing that cognitive impairment was present as a feature of OWS itself. While perhaps inadvertently pioneering research on OWSCI, the study also entailed many methodological issues that highlight the difficulty of generating well‐controlled data on OWSCI that manages the many variables and confounds inherent to this domain of research. For instance, many of the subjects had polyaddictions involving sedatives and alcohol (in addition to OUD). Before detox, when their cognition was initially assessed, their possible acute intoxication involving heroin and other substances was not accounted for. Then, subjects undergoing/finishing detox were cognitively tested at different time points in their OWS course (from early to late‐OWS; indicating that the authors were not attempting to control or track this variable). They were also treated by differential opioid versus non‐opioid detox regimens, and OWS itself was not scaled at any point. The overall findings that cognition improved in patients with heroin addiction (and approached the performances of healthy controls) during OWS compared to active use suggested that OWS does not produce cognitive impairment that is greater than what active heroin and polysubstance use entail.

### Pioneering cognitive testing in precipitated OWS: Kanof et al. (1992; United States)

In 1992, Kanof et al. published the first study designed to investigate cognition in acute precipitated OWS. This was done in a highly controlled way using iv naloxone (NLX) to induce a brief phase of precipitated OWS in US veterans maintained on methadone. Cognition after NLX was compared to a different session (using a randomized, cross‐over, repeated measure design) in which patients were given saline (placebo).^38^ This study, although designed somewhat like Guerra et al., by repeating cognitive testing during a state of active opioid receptor agonism versus a state of withdrawal, entailed tighter experimental control of clinical variables and subject selection. Patients entering the study were stable in methadone maintenance at standardized doses (24 mg/day); they were not engaging in the chaos of street heroin use (as in Guerra et al.) or polysubstance intoxication/withdrawal. Also, in Kanof, they had cognitive testing at well controlled and tightly consistent timepoints, just after receiving either NLX or placebo. This study also innovated the deployment of OWS measures including the Subjective Opioid Withdrawal Scale (SOWS), the Objective Opioid Withdrawal Scale, and associated autonomic and affective measures, in close temporal proximity to cognitive measures. Notably, this study (uniquely among the 7 core papers) produced a negative result in defining cognitive impairment during OWS that otherwise generated some dysphoria and other subjective/objective signs of OWS. However, the authors noted that the precipitated OWS episodes were relatively short in duration and shallow compared to what happens over the course of enduring a more complete, naturalistic 1–2‐week‐long OWS course.

Comparing Guerra and Kanof portends the methodological diversity that would continue across the subsequent five studies looking at OWSCI highlighting the complexity of interpreting this literature in summation. The degree to which studies include patients with real‐world comorbidities versus focusing on more scientifically pure subject pools; how the studies are designed (e.g., as repeated measures vs. cross‐sectional studies); when they test cognition (in the use/abstinence or post withdrawal cycle); if, how and when they assess the magnitude of OWS; and exactly what cognitive measures they use, would all be highly variable in subsequent studies. However, the emergence of a single widely accepted and well‐studied OWS measurement tool (the Clinical Opioid Withdrawal Scale; COWS), from among at least 18 different OWS scales that have been invented in the last 100 years,^39^ would bring more uniformity of OWS measurement across studies (3 of 5 would use the COWS). Also, multiple studies would become somewhat more consistent in cognitive measure utilization (e.g., using the Wisconsin Card Sort Test (WCST) in three of five subsequent studies). As summarized next, the five studies on OWSCI conducted after Kanof, all published this century, depart from Kanof by confirming and characterizing the existence of OWSCI at some point(s) in the course naturalistic OWS.

### Cognitive testing in natural OWS in methadone maintenance: Lyvers et al. (2003; Australia)

In 2003, Lyvers and Yakimoff cross‐sectionally compared cognition in methadone maintenance patients right before their daily methadone dosing (e.g., presumably in a low grade/early state of OWS at 24 h since last dose) versus patients having just received their Methadone dose.^40^ In this study, the pre‐dose (early OWS) patients showed greater cognitive impairment compared to those freshly dosed. This study did not utilize a repeated measure design, and the magnitude of OWS was not assessed.

### Cognitive impairment and duration of detoxification treatment: Rapeli et al. (2006; Finland)

In 2006, Rapeli et al. published their study of cognition during a detoxification‐withdrawal treatment protocol, measured cross‐sectionally in patients at mid‐ to late‐OWS (as confirmed by use of the Short‐Opioid Withdrawal Scale) compared to healthy controls.^41^ As with the original Guerra study, these patients had a real‐world polyaddiction comorbidity profile (operating as a potential confound) in which substantial proportions of the sample also had sedative‐hypnotic and cannabinoid use disorders they were also withdrawing from. Here, there was not a standardized timepoint of cognitive performance measurement (e.g., since the time of last opioid use) allowing the duration of OWS to become an independent factor. Leveraging this design feature, Rapeli was the first to show that OWSCI severity may depend on when in the course of OWS a patient may be, with mid‐OWS patients showing greater cognitive impairment compared to late‐OWS patients (both compared to a healthy control group). These patients were not previously in opioid maintenance treatment, and unfortunately their OUD histories (types/patterns of uncontrolled use) were not specified.

### Pioneering multi‐group comparisons for assessing OWSCI: Saroj et al. (2020; India)

Fourteen years after Rapeli, Saroj et al. reported their cross‐sectional study of cognition in OUD patients (unspecified type) undergoing OWS, innovating an interesting set of three control groups for their characterization of OWSCI.^42^ They examined cognition in early to mid‐OWS (treated with clonidine), compared to (i) healthy controls versus (ii) patients not in OWS, and stable on BUP versus (iii) patients not in OWS, and in opioid‐free recoveries maintained on NTX. This study sample was fairly purified from other SUDS with only nicotine use being allowed and no significant active psychiatric or medical morbidity. An interesting parameter the investigators also set in this study was to exclude patients in moderate to severe OWS (COWS scores > 12). This maneuver would presumably limit variability and extremes of poor cognitive performance in OWS. Even so, the study found that OWS patients showed worse cognitive performance among all groups overall, with BUP and NTX groups performing closer to HCs and NTX group performing closest to HCs. These results appear to support the existence of OWSCI as a transient phenomenon independent from other cognitive deficits of the OUD spectrum. OWSCI was found to not only correlate with OWS severity as measured by COWS, but it was more pronounced than the impairment found in the BUP group (in which high tolerance, and stable BUP compliance would negate acute opioid effects on cognition). The OWS group showed some greater impairment than the NTX group, while all three OUD groups showed some worse performance compared to HCs, consistent with opioid addiction carrying some mild cognitive impairments that may be more pronounced in OWS.

### Pioneering multi‐time point comparisons across OWS: Kaur et al. (2023; India)

Kaur et al. in 2023 reported their OWSCI study, which innovated the most comprehensive repeated measures design to date, where patients got cognitive testing across 3 time points: Before OWS, late‐OWS, and long after (8 weeks from last opioid).^43^ As with Saroj, this study also examined a population that was relatively free of other SUDs and mental health comorbidities (except nicotine), and they excluded patients in high withdrawal, but allowing a higher degree of OWS (COWS < 25). In this study, unlike Saroj, the patients using opioids were not stable in opioid maintenance treatment (i.e., their baseline (BL) cognition was measured while they were in a chaotic opioid use pattern), and OWS was treated (in unclear combinations) with clonidine and zolpidem (a benzoid with acute cognitive effects).^44^ In this study, the patients in active use (BL) showed the most severe cognitive impairment, which resolved considerably, but was still present in late‐OWS, although resolved further in sustained opioid‐free recovery (8 weeks later) when patients were maintained on NTX.

### Innovating biomarkers in a multi‐time point OWS‐cognitive study: Grant et al. (2024; Russia)

The most recent OWSCI study, and the first to include biological measures along with OWS measures (using COWS), was published by Grant et al. in 2024. This study was also unique in that it focused on OUD (involving heroin and/or methadone) in people with HIV infections (not on antiretroviral therapy) who were in a detoxification unit.^30^ As in Kaur, this study used repeated measures of cognition in early and mid‐late‐OWS (inpatients treated with clonidine) and after OWS (3–5 weeks post last opioid use). Some comorbid addictions (not cannabis or cocaine) were in the sample. As in Kaur and Saroj, cognitive impairment was found during OWS but was relatively resolved after OWS. Interestingly, this study was the first to characterize a biological correlate to OWSCI: HPA axis activation, measured as the cortisol/DHEA (dehydroepiandrosterone)‐sulfate ratio, was increased during OWS and correlated with greater cognitive impairment and worse mood disturbance. The extent to which these associations may be generalizable to OWS patients without HIV remains unknown as untreated HIV patients have abnormal vulnerability to immune compromise and HIV‐associated neurocognitive disorders (HANDs).

## DISCUSSION

This review of studies characterizing OWSCI within the first 15 days of opioid abstinence describes a small but growing database depicting OWSCI as a measurable dynamic phenomenon that exists apart from cognitive impairments associated with opioid intoxication or opioid addiction. However, OWSCI should be understood as being superimposed on these OUD spectrum causes of cognitive impairments, and a wide range of other concurrent factors and comorbidities of opioid addiction which can make its measurement challenging. Only one of seven studies did not find evidence for OWSCI, and it was arguably the methodologically weakest study, in which OWS was brief, mild, and artificially induced with NLX.^38^ Conversely, the three studies involving repeated measure designs of subjects undergoing a full course of naturalistic OWS^30^, ^37^, ^43^ showed that OWSCI evolves and generally improves with OWS resolution. Comparisons of cognition during OWS were made with cognition during active untreated street‐opioid use conditions in two studies,^37^, ^43^ which both showed greater impairment during uncontrolled use than late‐OWS. This contrasts with what we see in studies finding OWSCI in patients on BUP or methadone maintenance^40^, ^42^ in which OWSCI appears relatively more severe compared to cognition during stable opioid maintenance treatment. It is reasonable to expect that these contrasts are real and replicable since measuring cognition during chaotic street use of high potency opioids is expected to involve assessments when patients are experiencing higher amplitudes of intoxication/withdrawal states involving opioids and other drugs, generating much higher degrees of cognitive impairment than what is seen with stable opioid maintenance. But even for patients in maintenance treatment on long‐acting methadone, there is a mild OWSCI that occurs 24 h after dosing, compared to immediately after dosing.^40^ These findings suggest how the detection and measurement of OWSCI is very dependent on the choice of comparisons.

The action of co‐occurring SUDs and withdrawal states involving non‐opioid drugs that are comorbid with OUD/OWS (that can also cause cognitive impairment) has not been directly measured or parsed out in the OWSCI literature. This literature also varies considerably in how studies allow for these comorbidities. This highlights a pervasive dilemma in all clinical SUD research, where investigators must strike a balance between achieving scientific purity and controlling confounds that can short‐change our understanding of real‐world conditions and mainstream patients who usually have multiple addictions and/or comorbid mental illness(s). Four of the seven studies were quite inclusive of patients with active non‐opioid drug use, addictions, or withdrawal syndromes, apart from nicotine,^30^, ^37^, ^40^, ^41^ and of course, virtually all of them were agnostic as to nicotine use without reporting on use of nicotine replacement therapies, for example, during OWS treatment. This literature is also quite diverse and somewhat vague on the use of OWS treatment medications that could also have cognitive effects, including benzoid drugs, antihistamines, or opioid tapering schedules. Given this complexity, the acronym OWSCI seems apt with an emphasis on “syndrome” in which it is understood that a wide range of comorbid‐interactive neuropsychiatric and pharmacological variables are often dynamically in play to give rise to cognitive impairment in OWS. This complexity is similar to what we see in the Neonatal Abstinence Syndrome (NAS) literature, which, although focused on preventing and treating OWS in babies, has shown that a very wide range of co‐occurring variables, beyond mom's opioid histories, including non‐opioid addictive drug use or psychoactive medication treatment, can impact risk and severity of NAS in babies.^45^ As for future OWSCI studies, it seems clear that the experimental conditions chosen to capture OWSCI could greatly impact OWSCI's characterization.

The form(s) of cognition shown to be impacted in OWSCI is one such factor that depends on the types of cognitive tests used across this literature. Different measures used in this literature (Table 3) have variously identified impairments in memory, attention, and processing speed. The most consistently used cognitive measure has been the WCST, appearing in three of seven studies^40^, ^42^, ^43^ with two showing significant OWSCI. The WCST has been used to investigate cognitive impairment in schizophrenia, and with subanesthetic doses of the non‐competitive glutamate NMDA‐receptor blocker ketamine. It is sensitive for detecting a wide range of structural or neurochemically induced connectivity disruptions involving the Prefrontal Cortex.^46^ Consistent with this evidence, functional neuroimaging has captured data on animals and humans who are in NLX‐precipitated OWS. In rats, NLX‐precipitated OWS produces fMRI signal intensity changes across hippocampal and several primary sensory, poly‐modal association, and limbic cortices including Prefrontal Cortical regions.^47^ In humans, NLX‐precipitated OWS produces changes in neural activity across basal ganglia, cortical sensory and cortical limbic areas including PFC regions, some of which are correlated to the degree of OWS severity.^48^

Taken together, the OWSCI literature and the neuroimaging data suggest that OWS produces a degree of cortical–subcortical discoordination and/or change in axodendritic communication that underpins both classic OWS (its subjective sensory, affective and objective autonomic‐somatic components) and objectively measurable cognitive effects in OWSCI. To the extent that this dysfunction involves the PFC network and its connectivity with subcortical‐centers governing motivation and reward‐based decision‐making, future directions of OWSCI research might consider examining cognition pertaining to decision‐making and impulse control. There is already evidence that a state of “opioid deprivation” in humans (which may or may not entail OWS) increases decisional impulsivity as measured by delay discounting.^49^

### Limitations of the literature search and available literature

Our primary electronic database searches were limited to crosses of just three general umbrella terms, when in fact there are scores of more specific terms for opioids (e.g., “heroin”), cognitive impairment (e.g., “deficiency”), or cognitive skills (e.g., “working memory”). However, the number of permutations of crosses and lists of items that could result from such an exhaustive utilization of terms would be intractable. In addition, we noted that the searches that we did manually peruse, especially in Google Search, did capture studies that included these term variations anyway. Also, given the clearly small size of this budding literature, in which the target papers were quite accurate in citing preceding target papers, the bibliographic searches seemed key and gave us confidence that our electronic database searches were more than adequate. Our search criteria (e) requiring cognitive measurement at 1–15 days from abstinence was chosen at the risk of excluding some studies that were capturing OWSCI (although not clearly stating the timing from last opioid, or involving patients who were in OWS while still on opioid tapers).

The compiled OWSCI literature itself remains underdeveloped and marred by considerable heterogeneities spanning differential allowances for comorbid SUDs, neuropsychiatric and medical illness, study designs, clinical contexts, inconsistencies of measurements or treatments for OWS (that may also impair cognition), selection of comparison groups, timing and forms of cognitive testing used during OWS, and underrepresentation of female subjects.

## CONCLUSION

The pioneering studies describing OWSCI is a small but growing international literature that suggests that much like acute intoxication, OWS can induce measurable transient cognitive impairments that resolve within weeks after last opioid use. The impairment magnitude and contrast with pre‐withdrawal measures depend on the pattern, type, and stability of opioid use before OWS, and other SUD comorbidities. Additional studies on OWSCI will be needed to better control for specific clinical variables and knowledge gaps that limit this literature, while achieving greater standardizations of design features and cognitive measures implemented. Besides gaining a better understanding of the clinical and medical‐legal implications of OWSCI, future OWSCI studies may also be important to developing new and more effective treatments for patients choosing sustained opioid‐free recoveries where full passage through OWS is uniquely necessary. Whether pharmacological exacerbation, neutrality, or reduction of OWSCI corresponds to a therapeutic benefit—helping patients tolerate OWS and achieve sustained opioid‐free recovery—remains to be determined.

## CONFLICT OF INTEREST STATEMENT

The authors declare no conflicts of interest.

## Data Availability

Data sharing not applicable to this article as no datasets were generated or analyzed during the current study.
